# Effects of Stocking Density on Growth Performance, Antioxidant Status, and Meat Quality of Finisher Broiler Chickens under High Temperature

**DOI:** 10.3390/antiox11050871

**Published:** 2022-04-28

**Authors:** Jiseon Son, Hee-Jin Kim, Eui-Chul Hong, Hwan-Ku Kang

**Affiliations:** Poultry Research Institute, National Institute of Animal Science, Rural Development Administration, Pyeongchang 25342, Korea; wltjs1206@korea.kr (J.S.); khj0175@korea.kr (H.-J.K.); drhong@korea.kr (E.-C.H.)

**Keywords:** heat stress, stocking density, antioxidants, meat quality, broilers

## Abstract

Environmental factors such as stocking density and high temperature can cause oxidative stress and negatively affect the physiological status and meat quality of broiler chickens. Here, we evaluated the effects of heat stress on the growth performance, antioxidant levels, and meat quality of broilers under different stocking densities. A total of 885 28-day-old male broilers (Ross 308) were subjected to five treatments (16, 18, 21, 23, and 26 birds/m^2^) and exposed to high temperatures (33 °C for 24 h) for 7 days. High stocking density (23 and 26 birds/m^2^) resulted in significantly decreased body weight (*p* < 0.01) and superoxide dismutase activity in the blood (*p* < 0.05) and increased (*p* < 0.05) rectal temperature and corticosterone. Additionally, the concentrations of heat shock protein 70 and malondialdehyde in the liver were higher in the 26 birds/m^2^ group (*p* < 0.05). Similarly, the 2,2-diphenyl-1-picrylhydrazyl radical scavenging activity of breast meat increased linearly as the stocking density increased (*p* < 0.05). There was increased shear force in breast meat at low stocking density (*p* < 0.01). Thus, lower stocking density can relieve oxidative stress induced by high temperatures in broilers and improve the antioxidant capacity and quality of breast meat during hot seasons.

## 1. Introduction

In the poultry industry, chickens are exposed to various environmental stress factors, including temperature, humidity, and stocking density. Meat quality and welfare are major concerns, and effects of stocking density and high temperature on broiler chicken production have been reported in earlier studies [1]. Chickens are bred for rapid production and growth, and this may make them vulnerable to stressful environments.

Owing to global warming, heat stress in hot environments has become a problem for the chicken industry worldwide [2]. Chickens are susceptible to high temperatures because of their inability to decrease their body temperature due to their feather cover and limited sweat glands [3]. At high temperatures, chickens increase their water intake, pant, and spread their wings to cool their body temperature [4,5]. In addition, acute and chronic heat stress can adversely affect meat quality [6]. Therefore, continuous heat stress can induce diverse adaptation responses in chickens.

Stocking density is also an important management practice for the production and welfare of poultry. In broilers under heat stress in summer, higher stocking density (HSD) has been linked with lower behavioral trait scores, as well as problems such as scratches, footpad dermatitis, poor feather cover and cleanliness, and bruising [7]. HSD is frequently practiced to enhance profitability, as it results in higher chicken production per fixed-stocking area [8]. However, it has been reported that heat stress from high temperatures may exacerbate problems related to overcrowding in broilers [8]. Different stocking densities are practiced depending on the country or rearing system [9]. In Korea, stocking density standards for broilers are determined according to the housing facilities and management enforced by the decrees of the Livestock Industry Act, Presidential Decree No. 30974. These standards state that the stocking density of broilers is 39 kg/m^2^ in windowless houses.

High stocking density and high temperature stress can create a poor quality environment and cause oxidative stress in broilers [8,10]. Oxidative stress can induce a negative physiological status and oxidative damage in lipids, nucleic acids, and proteins in tissues [11]. To reduce heat stress in chickens, studies have investigated the effect of feed additives, nutrients, and stocking densities [5,6,8,11]. However, few studies address the effects of stocking density and heat stress in chickens. Particularly, high stocking density and heat stress can adversely affect broilers (e.g., high mortality, low growth performance, low meat quality, and stress), which reduces production [12]. The objective of our study was to investigate the effects of stocking density on growth performance, breast meat quality, and antioxidant and stress indicators in broiler chickens under high-temperature conditions.

## 2. Materials and Methods

### 2.1. Animals and Experimental Design

A total of 885 28-day-old male Ross 308 broilers were weighed and randomly as-signed to 5 different groups of 16, 18, 21, 23, or 26 birds per 1.7 m^2^ pens floor. Each of treatments consisted of five replicates, respectively, of 27, 31, 36, 39 and 44 birds for the calculation of stocking density. The stocking density complied with the Korean standards, and 26 birds with a calculated stocking density of 1.5 kg were set as a control group. Each pen had one bell drinker and one feeder at the same location. The floor was covered with a 5 cm deep layer of rice hulls. All the chickens were raised at 32 °C ± 1 °C during the first week of the study; then the temperature was gradually decreased to 24 °C by the 27th day. Heat stress environmental conditions (33 °C ± 1 °C for 24 h) were introduced from days 28–35. Two gas heaters with a sensor thermostatic controller were used to maintain the high temperature, and were controlled to minimize the temperature difference of each pen. The average relative humidity, controlled by means of an electronic controller humidifier, was maintained at 55% ± 5%. The heat treatment was applied for seven consecutive days, and the temperature was monitored several times daily during the HS period in different locations of the pens to ensure a homogenous normal distribution of the treatments. The birds were provided with food and water ad libitum.

### 2.2. Growth Performance and Sample Collection

Body weight (BW), feed intake, body weight gain (BWG), and the feed conversion ratio (FCR) were recorded on days 27 and 35. On day 35, three birds from each pen were randomly selected and euthanized via CO_2_ asphyxiation for sample collection. Blood samples were collected from the wing vein and placed into serum separator tubes (SST; BD Bioscience, Franklin Lakes, NJ, USA). To estimate the serum biochemical parameters, corticosterone, and total superoxide dismutase (SOD) activity, blood samples were centrifuged at 3000 rpm at 4 °C for 15 min to separate the serum and were stored at −70 °C before analysis.

### 2.3. Rectal Temperature and Respiration Rate

Three broilers from each pen were randomly selected and the rectal temperatures of those birds were recorded using a digital thermometer inserted approximately 3 cm into the rectum for 30 s on days 0, 3, and 7 of the exposure to heat stress. The respiration rate was measured in three randomly selected birds per pen on days 0, 3, and 7 of exposure to heat stress conditions. The number of breaths taken over 30 s was quantified and expressed in breaths/min.

### 2.4. Blood Biochemistry, SOD Activity, and Corticosterone Concentration

Serum biochemical parameters, including total cholesterol, triglycerides, glucose, total protein, albumin, aspartate aminotransaminase (AST), alanine aminotransferase (ALT), creatinine, inorganic phosphorus (IP), and lactate dehydrogenase (LDH) were measured using an automatic biochemistry analyzer (AU480 Chemistry Analyzer, Beckman Coulter Inc., Brea, CA, USA).

SOD activity in serum was assayed using the SOD assay kit WST (Dojindo, Tokyo, Japan). Absorbance at 450 nm was recorded using a microplate reader (Epoch2, Biotek Instruments, Winooski, VT, USA), and the superoxide inhibition rate was calculated according to the manufacturer’s protocol. The superoxide inhibition rate was used to calculate the percentage of inhibition of the WST reduction. SOD activities were defined as a unit (U)/g meat that was expressed as the amount of SOD of meat extract that inhibited the reduction of WST by 50%.

Corticosterone levels were measured using a commercial enzyme-linked immunosorbent assay (ELISA) kit (ADI-900-097, Enzo Life Science, Inc., Farmingdale, NY, USA). ELISA was performed according to the manufacturer’s protocol.

### 2.5. Liver Antioxidant Status

Liver tissue was homogenized in a tissue buffer containing a protease inhibitor cocktail (GenDEPOT, Barker, TX, USA) to estimate heat shock protein 70 (HSP70) and malondialdehyde (MDA) contents. The protein concentration in the supernatant obtained after centrifugation was quantified using a bicinchoninic acid protein assay kit (Sigma-Aldrich, St. Louis, MO, USA).

The concentration of HSP70 in the liver was assayed using a chicken HSP70 ELISA kit (Cusabio Life Science, Wuhan, China) according to the manufacturer’s instructions.

Liver MDA (lipid peroxidation) levels were analyzed using thiobarbituric acid-reactive substances, as described previously [13]. The MDA content was calculated from a standard curve of 1,1,3,3-tetraethoxypropane and expressed as nmol MDA per mg protein in the liver.

### 2.6. Breast Meat Antioxidant Status

The 2,2-diphenyl-1-picrylhydrazyl (DPPH) radical scavenging activity was analyzed using the supernatant collected from breast meat (*Pectoralis major*), according to [14]. Five grams of meat was homogenized in 25 mL of distilled water. Then, 10 μL of breast meat homogenate was mixed with 90 μL distilled water and 100 μL of 0.2 mM DPPH solution and was kept at room temperature (in a dark room) for 30 min for the reaction. Absorbance was read at 517 nm using a microplate reader (Epoch2, Biotek Instruments, Winooski, VT, USA) using distilled water as a blank. The DPPH radical scavenging activity was calculated as follows: DPPH radical scavenging activity (%) = (1 − (absorbance of sample/absorbance of control)) × 100.

The thiobarbituric acid reactive substance (TBARS) value was measured after storage at 4 °C for 7 days using the method described in [15]. Five grams of meat was homogenized in 15 mL of distilled water and 50 μL of 10% butylated hydroxyl anisole solution. Then, 1 mL of the homogenate was mixed with 2 mL of 20 mM 2-thiobarbituric acid solution (in 15% trichloroacetic acid solution). The mixture was heated in a water bath (80 °C) for 15 min and cooled on ice for 10 min. It was then centrifuged at 2000× *g* for 10 min. The absorbance of the supernatant was measured at 531 nm (Epoch2, Biotek Instruments Inc., Winooski, VT, USA). The TBARS value was expressed as milligrams of malondialdehyde per kilogram of meat (mg MDA/kg meat).

### 2.7. Breast Meat Quality

For breast meat (*Pectoralis major*), quality, pH, cooking loss, water holding capacity (WHC), and shear force were analyzed following the methods described in [16]. The pH of the right breast meat was measured using an Orion 230A pH meter (Thermo Fisher Scientific, Waltham, MA, USA) as follows: 10 g of meat was homogenized with distilled water (90 mL) using a homogenizer (Polytron PT-2500E; Kinematica, Lucerne, Switzerland) for 15 s, and the shear force of the breast meat was measured using a texture analyzer TA1 (Lloyd Instruments, Fareham, UK) with a V blade. To measure cooking loss, the samples were weighed, placed in a plastic bag, and then cooked in a water bath (80 °C) for 20 min. After cooking, the samples were cooled at room temperature for 10 min, and cooking loss was calculated as the percentage of loss in relation to the initial weight. The left breast meat was used to measure water holding capacity (WHC). Breast meat (0.5 g) was placed on a Millipore Ultrafree-MC (Millipore, Bedford, MA, USA), boiled at 80 °C for 20 min, cooled to room temperature, and then centrifuged at 4 °C for 10 min at 2000× *g* using a centrifuge machine to measure water loss.

### 2.8. Statistical Analysis

All data were analyzed via one-way analysis of variance (ANOVA) and polynomial contrast (linear and quadratic) using SAS software (version 9.4; SAS Institute Inc., Cary, NC, USA) to determine the effect of the stocking density level. All statistical analyses of results were performed with an individual chicken as the experimental unit and analyses including feed intake and FCR were performed with a pen as the experimental unit. For weight and weight gain, *n* = 135, 155, 180, 195, and 220; for feed intake and FCR analysis, *n* = 5; for rectal temperature and respiration rate, *n* = 15; and for plasma parameters, liver antioxidant, and meat quality, *n* = 15. Statistical differences among the treatments were grouped using Tukey’s new multiple range test. Differences were considered statistically significant at *p* < 0.05.

## 3. Results

### 3.1. Growth Performance

As shown in Table 1, the BW and BWG values in the highest stocking density group were significantly (*p* < 0.01) lower than those in the low stocking density groups (18 and 16 birds/m^2^). The results for the feed intake were not significantly different between the groups, although feed intake showed a linear increase as the stocking density decreased (*p* < 0.05). There was no significant difference in FCR among the groups.

### 3.2. Rectal Temperature and Respiration Rate

The rectal temperature and respiration rate of broilers on the heat exposure day are shown in Table 2. Before heat stress, there was no significant difference in rectal temperatures among the groups. After chronic heat treatment for 31 and 35 days, the rectal temperatures of birds in the 16 birds/m^2^ group were lower than those in the 26 birds/m^2^ group; the rectal temperature showed a linear increase as the stocking density increased (*p* < 0.05). The respiration rate did not differ among the groups on day 28. However, after exposure to heat stress for 31 days, the lowest respiration rate (*p* < 0.05) was found in the 16 birds/m^2^ stocking density groups, and this continued to linearly decrease thereafter (*p* < 0.05).

### 3.3. Blood Biochemistry, SOD Activity, and Corticosterone Concentration

Table 3 shows the blood biochemical parameters of the experimental groups. The level of glucose exhibited a linear decrease as the stocking density increased (*p* < 0.01). The albumin contents showed no differences between the groups, although there was a linear increase (*p* < 0.05) with an increase in the stocking density.

**Figure 1:**
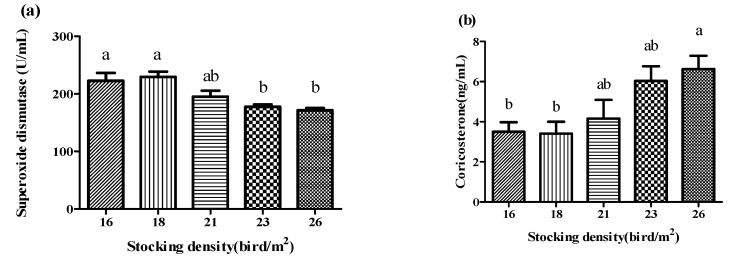
Effect of stocking density on superoxide dismutase (SOD) activity (**a**) and corticosterone (**b**) in the blood of broiler chickens under high temperatures (*n* = 15). SOD activity: linear, *p* < 0.001; quadratic, *p* = 0.800. Corticosterone: linear, *p* < 0.001; quadratic, *p* = 0.344. Bars with different letters (a,b) differ significantly across all groups (*p* < 0.05).

The SOD activity and corticosterone concentrations are shown in Figure 1. The activity of SOD linearly decreased with increased stocking density (*p* < 0.01) and was significantly lower (*p* < 0.01) in the 23 and 26 birds/m^2^ groups. The concentration of corticosterone was significantly higher (*p* < 0.01) in the 26 birds/m^2^ group than in the 16 and 18 birds/m^2^ groups. With increasing stock density, the corticosterone concentration increased linearly (*p* < 0.01).

### 3.4. Liver Antioxidant Status

**Figure 2:**
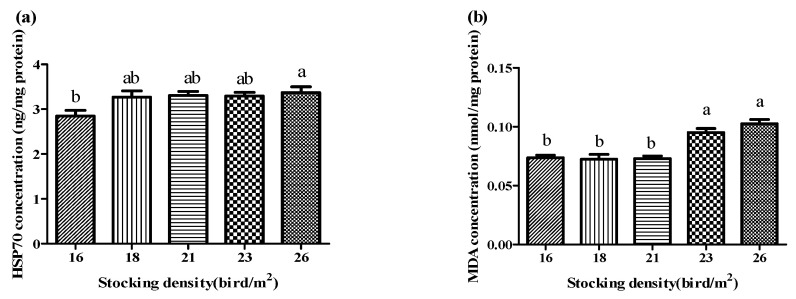
Effect of stocking density on heat shock protein 70 (HSP70) concentrations (**a**) and malondialdehyde (MDA) concentrations (**b**) in the livers of broiler chickens under high temperatures (*n* = 15). HSP70: linear, *p* < 0.01; quadratic, *p* = 0.103. MDA: linear, *p* < 0.001; quadratic, *p* < 0.01. Bars with different letters (a,b) differ significantly across all groups (*p* < 0.05).

Figure 2 shows the concentrations of HSP70 and MDA in the livers of the chickens. HSP70 decreased linearly (*p* < 0.05) with decreasing stocking density; the lowest concentration (2.8 ng/mg protein) was detected in the 16 birds/m^2^ group (*p* < 0.05). The level of MDA in the liver increased linearly (*p* < 0.01) and quadratically (*p* < 0.01) with increasing stocking density. The MDA level was significantly higher (*p* < 0.01) in the 23 and 26 birds/m^2^ groups than in the lower stocking density groups (16, 18, and 21 birds/m^2^).

### 3.5. Breast Meat Antioxidant Status

**Figure 3:**
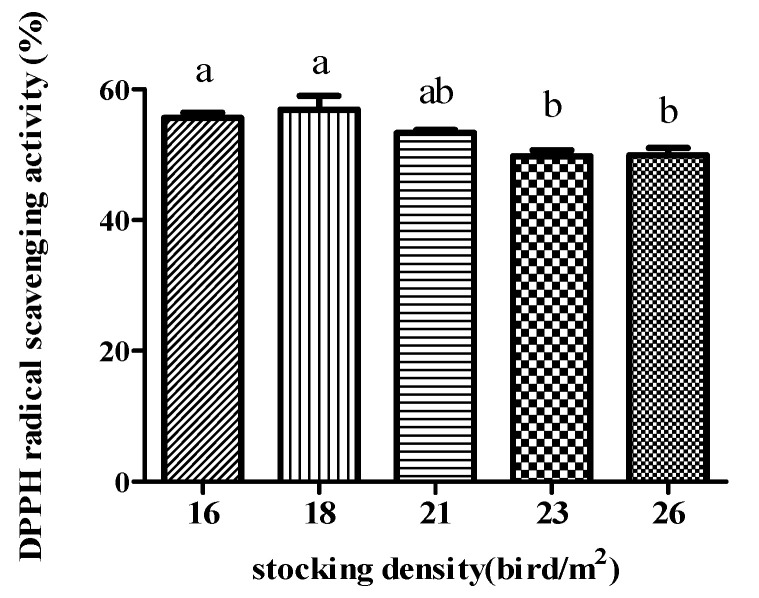
Effect of stocking density on 2,2-diphenyl-1-picrylhydrazyl (DPPH) radical scavenging activity in the breast meat of broiler chickens under high temperatures (*n* = 15). Linear, *p* = 0.001; quadratic, *p* = 0.616. Bars with different letters (a,b) differ significantly across all groups (*p* < 0.05).

DPPH radical scavenging activity is shown in Figure 3. The breast meat antioxidant status increased with decreasing stocking density (*p* < 0.01). It was significantly increased (*p* < 0.01) in the 16 and 18 birds/m^2^ groups compared with the 23 and 26 birds/m^2^ groups.

The TBARS content of breast meat during storage is shown in Table 4. The concentration of MDA for the 26 birds/m^2^ group was significantly higher (*p* < 0.01) than that of the 16, 18, and 21 birds/m^2^ groups after 5 days of storage. Increasing stocking density resulted in linear (*p* < 0.01) and quadratic (*p* < 0.05) effects after 5 days of storage. After storage for 7 days, the TBARS content of breast meat showed a decreased linear response (*p* < 0.01) with decreasing stocking density.

### 3.6. Breast Meat Quality

The breast meat quality results are presented in Table 5. The pH did not differ between the treatments.; however, the WHC was significantly higher (*p* < 0.01) in the 23 birds/m^2^ groups than in the 16 birds/m^2^ groups. The WHC of breast meat increased linearly (*p* < 0.01) and quadratically (*p* < 0.05) as the stocking density increased. However, the cooking loss of breast meat was not affected by stocking density. The shear force of breast meat showed a positive linear response (*p* < 0.01) with decreasing stocking density.

## 4. Discussion

Heat stress is known to exacerbate oxidative stress when combined with high stocking density. The feed intake of chickens reportedly decreases because of the alleviation of additional metabolic heat responses under heat stress [5,17]. When chickens are exposed to chronic heat stress, a decrease in BWG is associated with decreased feed intake and poor nutrient digestibility and absorption [18]. Previous studies have reported that under heat stress, high stocking density had an adverse effect on BWG, compared with low stocking density [8,19,20]. Additionally, several studies have revealed that increased stocking density is more stressful and is accompanied by reduced BW in chickens [21]. Increasing the stocking density from 20 to 50 birds/m^2^ was shown to decrease weight gains, owing to a linear decline in feed intake [22]. In addition, when stocking density was increased from low (28 to 37 kg/m^2^) to high (40 kg/m^2^), the feed intake decreased [23]. Our results are consistent with those of previous studies, showing that high stocking density negatively affects weight gain and feed intake. This suggests that high stocking density may both inhibit physical access to feeders and also cause birds to decrease their feed intake in order to maintain their body temperature under heat stress.

Rectal temperature and respiration rate are variables that are usually considered and used as the clear ones for estimating the physiological conditions of broilers [24]. Due to the lack of sweat glands, chickens spread their wings away from their bodies to increase the surface in contact with the air and pant to control body temperature in high-temperature environments [25]. High rectal temperatures and respiration rates suggest a physiological response under hot temperatures (i.e., respiratory alkalosis [26]). Elevated ambient temperature under heat stress led to increased rectal and body temperature in all groups. In addition, high stocking densities reduce airflow, impede body temperature dissipation, and make it difficult for chickens to cope with heat stress [27]. Our results show that as stocking density decreases, heat stress in broilers is alleviated, resulting in a decrease in rectal temperature and panting.

Chickens tend to undergo physiological changes as an adaptive mechanism in stressful environments [18,28]. In the blood chemical composition results, the blood sugar level was the lowest at a high stocking density, but the levels of total cholesterol and triglycerides did not show significant differences among the groups. Chronic heat stress reportedly decreases feed intake and results in poor nutrient digestibility and absorption in broilers [18]. Similarly, Salmonella-infected birds showed decreased glucose content because of low feed intake and absorption of nutrients [29]. In our study, the low blood sugar levels recorded might reflect reduced feed intake under high stocking density and absorption of nutrients during chronic heat. Birds reduce their feed intake and increase their fat reserves via high fat synthesis in the liver in response to heat stress; this negatively affects organ function [30,31]. Therefore, in our study we analyzed AST and ALT as indicators of liver damage and found no difference among the groups. Similarly, previous studies have shown that AST and ALT concentrations in the serum were not significantly different among groups [18,32]. However, some studies have demonstrated that heat stress increases the serum levels of AST and ALT in broilers [26,33]. The differences in these results may be attributed to the different experimental treatments. Total protein and albumin are released into the blood due to oxidative damage and stressful conditions [34]. Our results showed significantly higher total protein and albumin levels at high stocking densities than at low stocking densities, demonstrating that combined stress causes oxidative damage to muscles, resulting in high total protein and albumin levels.

Oxidative stress is induced by heat stress and stocking density, which can induce long-term corticosterone secretion. It also damages immunity and the antioxidant system, and causes muscle breakdown in broilers [35]. SOD is an important enzyme in the initial protection against reactive oxygen species (ROS) [36], and it can catalyze endogenous antioxidant enzymes [6]. In addition, SOD can inhibit excessive ROS accumulation in tissues and lipid oxidation in meat during storage [37]. MDA generated from lipid peroxidation is a highly reactive compound that is associated with oxidative stress, resulting in an increase in free radicals [38]. Acute heat stress increases the activity of antioxidant enzymes in response to oxidative stress [39,40] but chronic stress has been reported to decrease SOD activity and increase MDA content [41,42]. In our study, the corticosterone content was higher in the high stocking density group than in the low stocking density group under heat stress. Therefore, our study shows that broilers under HSD are more vulnerable to stress, which can reduce their growth performance. HSP expressed in the stress response is constitutively synthesized in cells, and prevents protein degradation and the repair of damaged cells under stress conditions [35].

HSP is usually plentiful in almost all organisms. HSP70 is constitutively synthesized in cells and prevents protein degradation and the repair of damaged cells under heat stress conditions [35]. The expression of HSP 70 is increased and upregulated in cells and tissues subjected to a variety of stresses, such as heating, stocking density, transportation, pre-slaughter, and others [43,44]. Several reports have showed oxidative stress to have a high correlation with the synthesis of HSP70, representing cell-induced HSP expression providing resistance to cell damage. HSP70 expression in acute heat stress has a negative correlation with the MDA content as an antioxidant system to protect the cells [45], but high HSP70 expression and MDA content indicate serious cell damage under chronic heat stress [45]. The HSP70 levels were elevated with the increase in stocking density (0.0578, 0.077, and 0.116 m^2^/bird) [46]. As expected, the 26 birds/m^2^ group showed the highest concentration of HSP70. From these findings, it can be concluded that the 26 birds/m^2^ group was exposed to higher heat stress than the 16 birds/m^2^ group. Furthermore, this result may promote thermal discomfort when associated with HSD.

Heat stress enhanced ROS production, including superoxide anions, peroxide, and free radicals, due to the increase in respiration. Furthermore, enhanced ROS levels in broilers under heat stress reduced antioxidant activity [47]. As a relevant indicator, DPPH free radical scavenging activity is widely used to evaluate antioxidant activity, the reduction of free radicals, and redox balance under different conditions [48].

SOD inhibits the production of free radicals under heat stress. One of the harmful free radicals is the superoxide anion radical (O_2_^·–^) [11]. Our results demonstrated that high stocking density was associated with decreased activity of SOD in the blood, which may be associated with the reduction of DPPH radicals in breast meat, and increased levels of MDA in breast meat during storage in breast meat.

High stocking density has been observed to drastically reduce carcass quality [23,44]. In terms of meat stability, pH is an important factor. A high pH can promote the growth of microorganisms, which can lead to rapid spoilage of meat [49]. However, in our study, there was no difference in relation to the stocking density. Our results show that heat and high stocking stress affected the reduction in shear force and increased WHC in breast meat. A previous study reported that heat stress increased the WHC of breast meat [44]. It has been suggested that chicken muscle degradation under environmental stress might be associated with an increase in proteolytic activity [50]. The shear force of muscle was shown to be reduced due to an increase in corticosterone under stressful conditions [51]. Corticosterone is a decisive factor in heat-stress-induced muscle catabolism [52], and induces protein degradation through the upregulation of atrogin-1, an animal muscle-specific ubiquitin ligase [52,53]. Low stocking density improves the meat shear force because of the increased motion of broilers [54]. Therefore, this suggests that the decreased shear force at high stocking density is evidence of a high stress status and lower activity level.

## 5. Conclusions

In conclusion, the results of our study demonstrated that increasing stocking density affected production and antioxidant systems and induced more oxidative stress under high-temperature conditions. HSD (26 birds/m^2^) induces a physiological oxidative stress response and reduces the meat quality compared to low stocking density (16 and 18 birds/m^2^). Future studies are required to evaluate the mechanisms of the relationship in which environmental stresses such as stocking density and heat affect physiological traits and meat quality. Nevertheless, these results could indicate that reducing stocking density under high-temperature conditions would have a beneficial effect on the production, antioxidants, and meat quality of broilers.

## Figures and Tables

**Table 1:** Effect of stocking density on performance of broiler chickens under high temperature during days 28–35.

Items	Stocking Density (Birds/m^2^)					SEM ^1^	*p*-Value	Linear	Quadratic
	16	18	21	23	26				
Initial BW (g/bird)	1345.5	1347.3	1353.3	1351.7	1356.8	7.322	0.993	0.664	0.990
Final BW (g/bird)	1988.6 ^ab^	2013.7 ^a^	1945.3 ^b^	1844.0 ^c^	1816.2 ^c^	8.361	0.010	0.001	0.327
BWG (g/bird)	643.1 ^ab^	666.4 ^a^	592.0 ^abc^	492.3 ^bc^	459.4 ^c^	26.017	0.010	0.001	0.348
Feed intake (g/bird)	1052.3	1067.3	1064.5	991.0	898.4	26.806	0.214	0.049	0.190
FCR	1.66	1.60	1.80	2.09	2.06	0.105	0.505	0.118	0.881

^1^ SEM, standard error of means. ^a–c^ Means with different superscripts in the same row were significantly different at *p* < 0.05. *n* = 135, 155, 180, 195, and 220 (body weight and body weight gain); *n* = 5 (feed intake and FCR). BW, body weight; BWG, body weight gain; FCR, feed conversion ratio. No mortality was observed during the experimental periods.

**Table 2:** Effects of stocking density on rectal temperature and respiration rate of broiler chickens under high temperatures.

Items	Stocking Density (Birds/m^2^)					SEM ^1^	*p*-Value	Linear	Quadratic
	16	18	21	23	26				
Rectal temperature (°C)									
d 28	40.5	40.6	40.6	40.7	40.6	0.040	0.515	0.112	0.434
d 31	42.0 ^b^	42.3 ^ab^	42.2 ^ab^	42.5 ^ab^	42.6 ^a^	0.111	0.013	0.001	0.835
d 35	42.4 ^b^	42.5 ^ab^	42.6 ^ab^	42.8 ^ab^	42.9 ^a^	0.077	0.021	0.002	0.946
Respiration rate (breaths/min)									
d 28	60.5	62.3	60.5	62.4	62.0	0.744	0.876	0.571	0.924
d 31	105.5 ^b^	110.9 ^b^	115.8 ^b^	126.6 ^ab^	140.0 ^a^	3.075	0.001	0.001	0.299
d 35	163.5 ^d^	178.3 ^cd^	181.8 ^bc^	193.8 ^ab^	201.3 ^a^	2.627	0.001	0.001	0.672

^1^ SEM, standard error of means. ^a–d^ Means with different superscripts in the same row were significantly different at *p* < 0.05. *n* = 15.

**Table 3:** Effect of stocking density on blood chemical composition of broiler chickens under high temperatures.

Items	Stocking Density (Birds/m^2^)					SEM ^1^	*p*-Value	Linear	Quadratic
	16	18	21	23	26				
Total cholesterol (mg/dL)	139.9	128.6	146.0	139.6	144.1	2.761	0.322	0.322	0.737
Triglyceride (mg/dL)	83.7	87.1	80.0	75.7	72.8	3.644	0.754	0.219	0.759
Glucose (mg/dL)	216.5	223.1	198.5	186.4	180.6	5.423	0.044	0.004	0.773
Total protein (g/dL)	3.2 ^ab^	3.1 ^b^	3.2 ^ab^	3.5 ^a^	3.5 ^a^	0.051	0.013	0.005	0.224
Albumin (g/dL)	1.33	1.23	1.34	1.37	1.38	0.017	0.063	0.046	0.057
AST (U/L)	356.8	343.8	367.7	344.4	327.5	8.963	0.706	0.379	0.479
ALT (U/L)	2.7	2.1	2.7	2.2	2.1	0.103	0.250	0.186	0.929
Creatinine (mg/dL)	0.2	0.2	0.2	0.2	0.3	0.003	0.603	0.388	0.258
IP (mg/dL)	7.8	7.9	8.0	7.1	7.6	0.161	0.367	0.235	0.796
LDH (mg/dL)	2508.4	2491.0	2474.0	2327.8	2181.1	67.111	0.553	0.106	0.511

^1^ SEM, standard error of means. ^a,b^ Means with different superscripts in the same row were significantly different at *p* < 0.05. AST, aspartate aminotransaminase; ALT, alanine aminotransferase; IP, inorganic phosphorus; LDH, lactate dehydrogenase. *n* = 15.

**Table 4:** Effect of stocking density on thiobarbituric acid (TBARS) in breast meat of broiler chickens under high temperatures during cold storage.

Storage Days (mg MDA/kg Meat)	Stocking Density (Birds/m^2^)					SEM ^1^	*p*-Value	Linear	Quadratic
	16	18	21	23	26				
0	0.307 ^B^	0.309 ^B^	0.307 ^B^	0.317 ^B^	0.318 ^B^	0.003	0.453	0.108	0.567
3	0.311 ^B^	0.312 ^AB^	0.318 ^AB^	0.318 ^B^	0.318 ^B^	0.002	0.694	0.210	0.565
5	0.320 ^ABb^	0.321 ^ABb^	0.321 ^ABb^	0.339 ^ABab^	0.370 ^Aa^	0.005	0.001	0.001	0.023
7	0.336 ^Aab^	0.328 ^Ab^	0.334 ^Aab^	0.350 ^Aa^	0.350 ^ABa^	0.002	0.004	0.001	0.103

^1^ SEM, standard error of the mean. ^A,B^ Means with different superscripts in the same column are significantly different at *p* < 0.05. ^a,b^ Means with different superscripts in the same row are significantly different at *p* < 0.05.

**Table 5:** Effects of stocking density on breast meat quality of broiler chickens under high temperatures.

Items	Stocking Density (Birds/m^2^)					SEM ^1^	*p*-Value	Linear	Quadratic
	16	18	21	23	26				
pH	5.87	5.91	5.90	5.85	5.86	0.014	0.685	0.544	0.402
Cooking loss (%)	15.8	15.2	15.4	14.8	15.5	0.254	0.821	0.660	0.419
WHC	49.4 ^b^	51.7 ^ab^	54.5 ^a^	54.1 ^a^	52.9 ^ab^	0.530	0.010	0.007	0.014
Shear force (N)	77.2 ^a^	74.7 ^a^	63.3 ^ab^	51.6 ^b^	49.8 ^b^	2.376	0.001	0.001	0.941

^1^ SEM, standard error of means. ^a,b^ Means with different superscripts in the same row were significantly different at *p* < 0.05. *n* = 15. WHC, water holding capacity.

## Data Availability

The data are contained within the article.
